# Clusterflock: a flocking algorithm for isolating congruent phylogenomic datasets

**DOI:** 10.1186/s13742-016-0152-3

**Published:** 2016-10-24

**Authors:** Apurva Narechania, Richard Baker, Rob DeSalle, Barun Mathema, Sergios-Orestis Kolokotronis, Barry Kreiswirth, Paul J. Planet

**Affiliations:** 1 Sackler Institute for Comparative Genomics, American Museum of Natural History, New York, NY 10024 USA; 2 Public Health Research Institute Center, New Jersey Medical School, Rutgers Newark, NJ 07103 USA; 3 Department of Pediatrics, Division of Pediatric Infectious Diseases, Children’s Hospital of Philadelphia & University of Pennsylvania, Philadelphia, PA 19104 USA; 4 Department of Biological Sciences, Fordham University, Bronx, NY 10458 USA; 5 Department of Epidemiology, Mailman School of Public Health, Columbia University, New York, NY 10032 USA

**Keywords:** Swarms, Flocking algorithm, Unsupervised clustering, Data mining, Horizontal gene transfer, Recombination, *Staphylococcus aureus*

## Abstract

**Background:**

Collective animal behavior, such as the flocking of birds or the shoaling of fish, has inspired a class of algorithms designed to optimize distance-based clusters in various applications, including document analysis and DNA microarrays. In a flocking model, individual agents respond only to their immediate environment and move according to a few simple rules. After several iterations the agents self-organize, and clusters emerge without the need for partitional seeds. In addition to its unsupervised nature, flocking offers several computational advantages, including the potential to reduce the number of required comparisons.

**Findings:**

In the tool presented here, Clusterflock, we have implemented a flocking algorithm designed to locate groups (flocks) of orthologous gene families (OGFs) that share an evolutionary history. Pairwise distances that measure phylogenetic incongruence between OGFs guide flock formation. We tested this approach on several simulated datasets by varying the number of underlying topologies, the proportion of missing data, and evolutionary rates, and show that in datasets containing high levels of missing data and rate heterogeneity, Clusterflock outperforms other well-established clustering techniques. We also verified its utility on a known, large-scale recombination event in *Staphylococcus aureus.* By isolating sets of OGFs with divergent phylogenetic signals, we were able to pinpoint the recombined region without forcing a pre-determined number of groupings or defining a pre-determined incongruence threshold.

**Conclusions:**

Clusterflock is an open-source tool that can be used to discover horizontally transferred genes, recombined areas of chromosomes, and the phylogenetic ‘core’ of a genome. Although we used it here in an evolutionary context, it is generalizable to any clustering problem. Users can write extensions to calculate any distance metric on the unit interval, and can use these distances to ‘flock’ any type of data.

## Background

Swarm intelligence describes cooperative behavior that results from a group of agents executing simple behavioral programs. The agents themselves are unsophisticated, but patterns emerge from the accumulation of pairwise interactions that help accomplish complex tasks necessary for the group’s survival [1]. Swarms are by definition leaderless, and agents therein are given no internal or external direction. Ant colonies, swarms of bees, shoals of fish, and flocks of birds all demonstrate this kind of behavior [2–5].

Because there is no central control, algorithms modeled on swarm intelligence excel at data-mining tasks where the goal is the discovery of unknown patterns in data. Early models of this type of behavior invoked the n-body problem in physics concerning the motion of celestial objects [6]. Later work integrated biological observations into this problem, particularly the importance of local information in determining global patterns of behavior [7–10]. The Reynolds flocking model [11] is one such example, designed specifically to simulate the characteristics of the coherent behavior of a flock of birds. Reynolds’ original goal was to bestow life-like animation on particles, which he termed “boids*,”* in motion pictures. In Reynolds’ flocking model, each boid in a simulation is a clone of every other; boids heed only their immediate surroundings as delimited by a radius of perception. They react to flockmates within this radius using a small library of simple behaviors that ultimately result in synchrony within the entire group. If boids are assigned bits of information, and if distances between these bits of information are easily computed, the flocking algorithm becomes suitable for unsupervised clustering.

In this study, we present Clusterflock, a method that aims to isolate groups (flocks) of genes with congruent historical signals. In our technique, boids represent orthologous gene families (OGFs), and the distance between them is measured as a simple test of phylogenetic congruence [12].

Phylogenetic incongruence is rampant in the evolutionary history of genes across most organisms in the tree of life [13–16], but the problem is particularly severe among bacterial genomes, where evolution proceeds through multiple mechanisms that destroy phylogenetic signals, such as recombination, *de novo* gene acquisition, and loss and duplication [17]. A central problem in microbial evolutionary biology is distinguishing these non-vertical mechanisms and separating vertical signals from horizontal ones produced by recombination and gene transfer. Whole genomes consist of thousands of genes with potentially varying histories, hence magnifying the analytical and computational complexity of this problem [18, 19]. The number of recombination events and the rates of gene transfer are often not known, and the inclusion of genes with different histories in the same analysis can lead to bias and error in phylogenetic trees. This kind of error is especially problematic in cases of large-scale recombination or sustained/concerted gene transfer between organisms that occupy the same habitat [20], situations that can lead to strong support for incorrect hypotheses.

A few algorithms have been proposed to address this phylogenetic problem in large whole-genome datasets. In general, such approaches rely on a two-step procedure: pairwise tests of phylogenetic incongruence between OGFs, followed by clustering to segregate the OGFs into congruent groups. A range of initial incongruence tests have been used, including such character-based incongruence measures as incongruence length difference (e.g., mILD [19]) and the likelihood ratio test (e.g., CONCATERPILLAR [21]), as well as topological measures (e.g., Conclustador [18]). For the clustering step, CONCATERPILLAR and mILD use agglomerative, hierarchical clustering techniques, whereas Conclustador uses k-means and spectral clustering algorithms. Hierarchical clustering techniques generally require a threshold value that defines the boundaries of groups, an assumption that can introduce bias or error. Spectral clustering, as implemented in Conclustador, and k-means algorithms require the prior estimation or specification of the number of clusters, which can lead to erroneous lumping or splitting. By contrast, Clusterflock does not require prior specification of distance thresholds or the number of groups.

We use incongruence length difference (ILD) in our analysis of the Clusterflock algorithm, but the algorithm can also be used with the likelihood ratio test or the topological incongruence measure in Conclustador [18]. Indeed, the flocking algorithm can cluster anything, given precalculated pairwise distances between all pairs of entities. In this study, we tested the flocking model against other clustering algorithms, such as multidimensional scaling (MDS), hierarchical clustering, and partitioning around medoids (PAM). In order to establish its use in practice, we used Clusterflock to analyze a well-studied example of massive genome recombination in *Staphylococcus aureus* clonal group ST239, involving nearly 20 % of the chromosome [22]. As in many phylogenomic datasets, large amounts of data were missing from the staphylococcal genomes that we analyzed, a circumstance that often limits the effectiveness of clustering algorithms [18]. While other techniques failed to segregate the recombined region of the ST239 genome, Clusterflock successfully distinguished the recombined genes from those in the recipient genome. We explored this resilience against missing data and rate heterogeneity through simulation ﻿analysis.

### Implementation

**Fig. 1:**
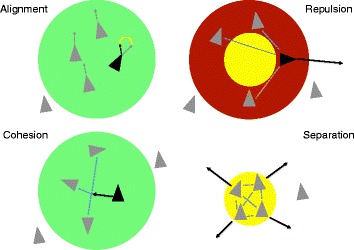
Flocking algorithm and rules. Agents (boids) are shown as *triangles*, interactions as *dashed lines*, and the radius of perception as differently colored *circles* depending on the vector considered. Alignment and cohesion reinforce flocking behavior while repulsion disrupts it. In alignment (*green*), a boid will adjust its trajectory to match congruent flockmates. In cohesion (*green*), a given boid moves toward the center of mass of all congruent flockmates within its field of vision. In separation (*yellow*), all boids maintain a minimum distance from one another regardless of whether they are congruent or incongruent. Cohesion, alignment, and separation are the core forces in Reynolds’ original flocking algorithm. We have added repulsion (*red*) which operates between an agent’s field of vision and the smaller, concentric circle describing its radius of separation. The magnitudes of alignment, cohesion, and repulsion are a function of the phylogenetic distance between the agents as described in the implementation

Reynolds’ original algorithm and our modifications to it are shown in Fig. 1. At the outset, agents (boids in the simulation) were assigned a random position and velocity in a two-dimensional field, normally set at one particle per square unit. Agents were then allowed to interact with one another. In the classic approach, each entity is influenced only by its local environment as given by a user-defined radius. For each agent, flockmates within this radius influence the calculation of three steering vectors that combine to alter the particle’s velocity: *Alignment*, *Cohesion,* and *Separation*. Agents tend to head in the average direction of their flockmates (alignment), move toward their average position (cohesion), and avoid crowding one another (separation). To accelerate the formation of flocks, we added *Repulsion* as a fourth vector, and designed it to operate between each agent’s field of vision (radius) and its radius of separation. In a clustering context, repulsion quickly separates agents with high relative distances, seeding flocks at an early point in the simulation. The calculation of the four vectors was iterated over all agents in the system for a user-defined number of frames.

In our adaptation, each particle was a set of aligned gene sequences (i.e., a phylogenetic matrix ﻿of an OGF﻿), and each interaction triggered a measurement (or a hash table lookup) of phylogenetic congruence between alignments in the pair. We modeled our congruence metric after the incongruence length difference (ILD) [23]:

$$ LD\kern0.5em =\kern0.5em \frac{L_{A+B}-\left({L}_A+{L}_B\right)}{L_{A+B}} $$

where *L*_*A+B*_ is the length of the Maximum Parsimony (MP) tree calculated when the two gene alignments were combined (i.e., concatenated), and *L*_*A*_ and *L*_*B*_ are the lengths of the trees calculated separately for each gene. An LD of zero indicated complete congruence (i.e., the gene trees were identical), whereas a positive LD indicated that the two OGFs had divergent phylogenetic topologies. To function as a distance metric on the unit interval, LD was normalized and, therefore, scaled between zero and one. We calculated single-gene parsimony trees and concatenated trees in PAUP* [24] using 100 heuristic searches, with random sequence addition, and tree bisection and reconnection.

Unlike other implementations [25, 26] we used the LD metric directly in our formulation of the steering vectors. This allowed the distance between any two OGFs to have a continuous effect on the simulation. More specifically, alignment and cohesion were calculated as the average velocity and position, respectively, of all flockmates within the field-of-view of any given agent (Fig. 1). This average was then modulated by two factors: the average LD of all flockmates, and a user-defined diminishment factor. The equation for alignment is as follows:

$$ v\kern0.5em =\kern0.5em \frac{1}{n}{\displaystyle \sum_x^n{v}_x}\bullet \frac{1-\frac{1}{n}{\displaystyle \sum_x^nL{D}_x}}{D} $$

where *v* is velocity driven by alignment, *n* is the number of flockmates, *v*_*x*_ is the velocity of flockmate *x*, *LD*_*x*_ is the LD for flockmate *x*, and *D* is the diminishment factor. In this scenario, as the average LD increased, the alignment or cohesion effect decreased. Similarly, as the diminishment factor increased, the alignment or cohesion effect decreased. The diminishment factor was intended as a layer of control, in order to provide the user the opportunity to up-weight or down-weight the alignment and/or cohesion vectors.

Separation and repulsion were treated somewhat differently, and were instead calculated through iterative displacement. Each agent within the separation distance updated the separation vector in turn in an attempt to double the distance between itself and its counterpart:

$$ v\kern0.5em =\kern0.5em v-\left({P}_x-P\right)\kern8.5em {\forall}_x\left\{1,..,n\right\} $$

where *v* is velocity driven by separation, and *P* and *P*_*x*_ are the positions of the agent in question and its flockmate *x*, respectively. Repulsion is enhanced separation that operates between the separation distance and the perception radius. It is directly proportional to the LD of flockmate X and a user-defined enhancement factor:

$$ v\kern0.5em =\kern0.5em v-\left(\left({P}_x-P\right)\times \left(L{D}_xE\right)\right)\kern5.5em {\forall}_x\left\{1,..,n\right\} $$

where *LD*_*x*_ is LD for flockmate *x*, and *E* is the enhancement factor. In this formulation, if the LD between any two OGFs was zero, the repulsion effect vanished. For positive LDs, repulsion was positive and proportional to the magnitude of the incongruence calculated. Increasing the enhancement factor could considerably magnify any existing repulsion.

**Fig. 2:**
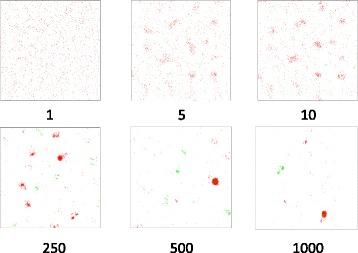
Snapshots from a *Staphylococcus aureus* simulation. Here, we show three early snapshots (1, 5, and 10) and three taken at intervals along a 1000-frame flocking simulation (250, 500, and 1000). Agents in green represent genes from the recombined region, whereas those in red are from the core genome. Specific parameters chosen here included: DIMENSIONS = 2500, BOUNDARY = 1, INIT_VELOCITY = 50, COHESION_FACTOR = 5, SEPARATION_DISTANCE = 5, REPEL_FACTOR = 10, ALIGNMENT_FACTOR = 5, ITERATIONS = 1000, RADIUS = 500, VELOCITY_LIMIT = 50, MINPTS = 20, XI = 0.15

The sum of the velocities derived from the cohesion, alignment, and separation/repulsion rules encodes the evolutionary distance information between an active agent and all its flockmates. When summed across all agents over all iterations, the OGFs converged on multiple evolutionary solutions, and the final frame of the simulation often isolated all congruent clusters. Figure 2 captures snapshots of this process. Seed clusters formed very early, and later moved to intercept one another. Congruent flocks absorbed, while incongruent flocks repelled, one another.

Clusterflock is a parameter-rich approach that allows the user fine-grained control over the steering of OGFs within the virtual space. In addition to the cohesion, alignment, and repulsion factors outlined here, other key parameters include the length of the virtual square that serves as the flight space, the radius of awareness about each agent, the initial velocity, the velocity limit, and the number of iterations. We found that a gene per square unit and a radial awareness of 10 % of the length of the flight space were sufficient to encourage efficient flock formation in most cases. Because velocity can increase quickly if left uncapped, limiting it to 2 % of the length of the virtual square was usually adequate for circulation.

In its original form, the algorithmic complexity of flocking was *O(n* ^*2*^ *)*. Each agent must spatially assess all other agents to determine the ones in its field of view (radius). We reduced this complexity by borrowing two heuristics, one from the world of videogames, and another from nature. In videogames requiring real-time calculation of the interactions among many particles, a spatial hashing structure [27, 28] reduces the number of required comparisons by binning particles into a discrete number of cells. Agents are sorted by their location, and only those in cells immediately surrounding the query are processed. In practice, the most significant savings attained by spatial hashing are accrued early in a simulation, when agents are evenly dispersed. As flocks begin to form, some cells are completely bereft, whereas others can contain thousands of congruent OGFs.

A more even and lasting heuristic is awareness. In nature, a member of a flock will not necessarily respond to *all* its immediate flockmates [10, 29]. Often, it responds to a mere subset, an approximation that rarely leads to unwanted perturbations or collisions because of the cumulative, emergent nature of the group’s motion as a whole. Capped by a user-input maximum awareness, a random sampling of flockmates for each gene is often sufficient to guide groups of flocks into distinct evolutionary classes. This heuristic can be important toward the end of a simulation, when many of the final flocks have formed. Groups of thousands of congruent individuals are not uncommon. By dimming each agent’s effective perception, only a fraction of these congruent individuals requires analysis.

Despite these shortcuts, flocking is a computationally expensive procedure. Without image/movie creation and analysis of the progress in cluster formation (k-means or OPTICS, see below), the flocking procedure alone requires, on average, 30 CPU seconds for a dataset containing 100 loci of 100 residues each across 10 taxa. Because we encourage 100 or more replicates, the total required CPU time per experiment for this hypothetical dataset would be just short of one CPU hour. By contrast, a single run of other clustering procedures, such as MDS, hierarchical clustering, and PAM, requires less than a CPU second to analyze this type of data. For Clusterflock, what is lost in terms of speed is gained in performance, as we show in the next section.

### In test: simulations and comparisons with prevalent clustering techniques

We simulated 100 locus datasets containing 100 residues per locus across 10 taxa in Seq-Gen [30] using JTT. Underlying these simulated proteins were anywhere from one to 25 generated topologies: in the case of one underlying topology, all 100 loci were modeled as congruent; in the case of 25 topologies, loci were randomly assigned to each tree without requiring that each tree be equally represented. Because missing data is a common problem in phylogenomics, we chose to model the effect of data sparsity on clustering performance. Taxa were randomly assigned as missing on a per locus basis at rates varying from 0 to 50 % in 10 % increments. Because rate heterogeneity is also a common problem, in a separate set of experiments with no missing data, we randomly assigned approximately half the proteins in each matrix to be 3X (1.5/0.5), 7X (1.75/0.25), or 19X (1.9/0.1) faster than their counterparts in terms of relative evolutionary rates [30]. For statistical power, we repeated dataset creation 10 times per topological condition across our missing data thresholds and rate heterogeneity multipliers, for a grand total of 2500 matrices.

In addition to using Clusterflock (100 replicates of 500 frames each), we analyzed the 100 proteins in each matrix using multidimensional scaling (MDS), hierarchical clustering, and PAM, by using the R packages [31] cmdscale, hclust, and pam, respectively. In the case of PAM, a k-medoids operation, and hierarchical clustering, users needed to provide an estimate of the expected number of clusters at the outset. In practice, providing this number is often difficult given the increasing complexity of modern phylogenomic datasets. By contrast, Clusterflock and MDS techniques spatially encode distance information between loci in order to allow for the emergence of distinct data categories.

**Fig. 3:**
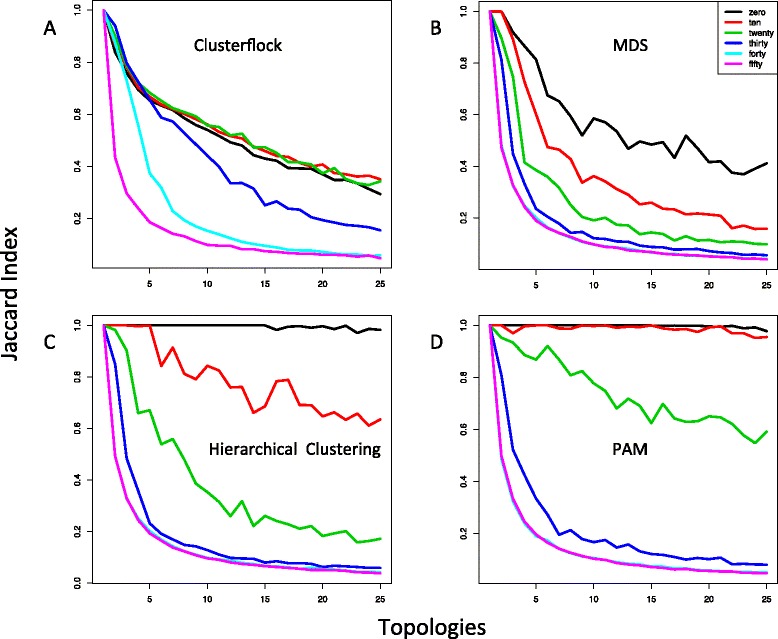
Simulating topological complexity and missing data. Four plots measuring the Jaccard Index (see *In test*) against increasing topological complexity and across increasing levels of missing data percentage (colored as indicated in the insert) are shown. Each curve in each plot, therefore, corresponds to a specific missing data condition. We compared four methods: (**a**) Clusterflock, (**b**) multidimensional scaling (MDS), (**c**) hierarchical clustering, and (**d**) partitioning around medoids (PAM). To compare the four techniques using similar methods, because the number of underlying topologies in each simulation was known, for Clusterflock and MDS, we used k-means to cluster the final spatial arrangement of loci and assign OGFs to topological groups

Figure 3 summarizes the results of these simulations. It shows the average Jaccard Index of all replicates against the number of simulated topologies across all data sparsity thresholds. Because we knew both the tree associated with each gene and the number of topologies in each simulation, we could use the Jaccard Index to measure the spatial groupings produced by Clusterflock and MDS clusters either by k-means clustering, which seeded its operation with our known number of clusters, or OPTICS [32], a method designed to identify unseeded clusters in spatial data regardless of their density. The Jaccard is defined as follows:

$$ J\kern0.5em =\kern0.5em \frac{TP}{TP+FP+FN} $$

where TP is the true positive, FP the false positive, and FN is the false negative, as judged by correct assignment of genes to a congruent tree topology group.

As expected, the performance of all four methods degraded with increasing topological complexity and data sparsity. No method performed well when 50 % of the data was missing, a condition that reduced the resolution of the LD metric such that the performance was only marginally better than random. Increasing the topological heterogeneity makes it difficult for any method to distinguish groups. However, Clusterflock was more robust against the effects of missing data than the other methods tested here. It also mirrored the performance of MDS as the evolution of the underlying genes became more complicated.

With the advantage of seeding, hierarchical clustering and PAM were superior in the special case where the number of topologies was known and used to guide cluster formation. As long as there was little missing data, these two methods were clearly superior of the techniques compared here. Surprisingly, hierarchical clustering and PAM performed poorly as data was removed.

We also tested CONCATERPILLAR, a technique that uses an agglomerative, hierarchical clustering technique that does not specify the numbers of seeds/groups, but requires a priori definition of a threshold for inclusion in a group. We tested CONCATERPILLAR for 2, 5, 10, 15, 20, and 25 underlying topologies at 0 % 10 %, 20, and 30 % missing data, and replicated each condition ten times. Given that CONCATERPILLAR is a hierarchical technique, we expected that it would perform similarly to the standard hclust function. In fact, CONCATERPILLAR was at least as good as hclust, and was more resilient against missing data up to a point (Table 1). Because its phylogenetic comparisons require the representation of all taxa, CONCATERPILLAR ceased to be useful at 40 % and 50 % missing data.

**Table 1:** Relative performance of CONCATERPILLAR and Clusterflock

	0 %	10 %	20 %	30 %
2	0.91/0.84	0.99/0.87	0.94/0.90	0.90/0.94
5	1.00/0.65	1.00/0.67	0.93/0.68	0.75/0.66
10	1.00/0.54	1.00/0.56	0.89/0.56	0.47/0.44
15	1.00/0.43	1.00/0.46	0.83/0.47	0.38/0.25
20	1.00/0.37	0.99/0.41	0.72/0.37	0.22/0.19
25	1.00/0.29	0.99/0.35	0.69/0.34	0.24/0.16

We tabulated Jaccard Indices of CONCATERPILLAR runs on the simulated datasets described at 0, 10, 20, and 30 % missing data (columns) across 2, 5, 10, 15, 20, and 25 simulated topologies (rows). For comparison, the average CONCATERPILLAR JI is shown alongside the average Clusterflock JI in each cell (CONCATERPILLAR|clusterflock). At 40 and 50 % missing data, CONCATERPILLAR failed to run (denoted by “NA”)

Because CONCATERPILLAR and Clusterflock may be used to address the same biological question, we have provided a head-to-head comparison of the two techniques (Table 1). In most situations with less than 20 % missing data, CONCATERPILLAR was superior. However, the two techniques are not strictly comparable, because CONCATERPILLAR requires setting an inclusion threshold whereas Clusterflock does not. Moreover, CONCATERPILLAR may not be computationally tractable in large genomic datasets. Although this is beyond the scope of this publication, it is possible that further implementations of Clusterflock that use strictly specified inclusion thresholds or other measures of phylogenetic incongruence might be more successful.

A more apt comparison for Clusterflock is MDS, which spares the user an initial estimation of the number of clusters and inclusion thresholds. Comparisons showed that increasing topological complexity lowered the Jaccard Index at approximately the same rate, as long as there was no missing data (Fig. 3 and b). The advantage of Clusterflock over MDS materialized once the data thinned. Still, when the underlying complexity was extreme, both these methods assigned loci correctly less than half the time, a result that highlights the difficulty in simultaneously solving both the number of clusters and their memberships.

**Fig. 4:**
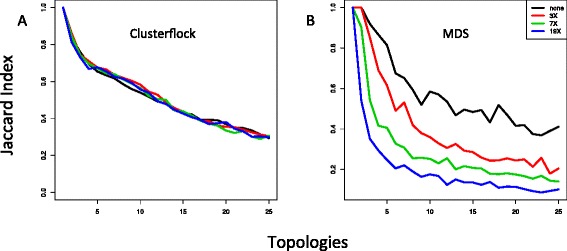
Simulating topological complexity and evolutionary rate heterogeneity. Here, we show two plots measuring the Jaccard Index against topological complexity and across four levels of the relative evolutionary rate: 1X (1/1), 3X (1.5/0.5), 7X (1.75/0.25), and 19X (1.9/0.1) (colored as indicated in the insert). Each curve in each plot therefore corresponds to a specific evolutionary rate condition. We compared two methods here: (**a**) Clusterflock and (**b**) multidimensional scaling

Figure 4 shows the effect of rate heterogeneity. MDS degraded rapidly with increasing rates of relative heterogeneity, whereas the strong performance of Clusterflock persisted across evolutionary rates. Therefore, up to a point, Clusterflock is resilient against both missing data and differing evolutionary rates. It performed equally well with 0, 10, and 20 % missing data, whereas MDS showed a quick collapse as information was removed. Further, diverging evolutionary rates among genes did not seem to affect the ability of Clusterflock to sort them into their respective topological groups.

**Fig. 5:**
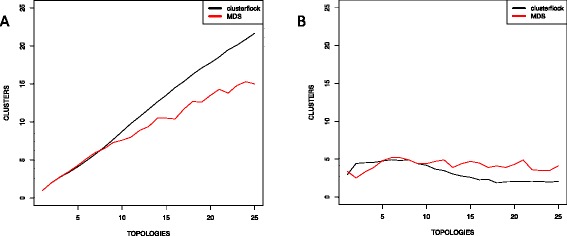
Clusters captured from simulated topologies. We plotted the number of topologies simulated against the number of clusters captured using either k-means (**a**) or OPTICS-based (**b**) density clustering of spatial coordinates from either Clusterflock or MDS

Although we favor the Jaccard Index as a more descriptive metric of clustering success, the absolute number of clusters returned relative to the number of topologies simulated was also revealing. For spatial arrangement by either Clusterflock or MDS, we used k-means to discover clusters for the number of topologies simulated. However, if the spread was insufficient, a few clusters went unpopulated. Figure 5a shows that in a k-means context, both Clusterflock and MDS found fewer congruent clusters than expected; however, it also shows that MDS suffered from a larger detection gap. Clusterflock thus seems to yield better separation between topologically distinct genes when heterogeneity is high.

Figure 5b shows the same data for OPTICS-based clustering. Clearly, the ultimate limitation to our chosen unsupervised clustering techniques is automated density-based detection of the final arrangement of points. More sophisticated k-means techniques, like elbow or silhouette [33], might be useful in this final step. However, for the most heterogeneous cases, visual inspection is probably the most reliable solution.

Real-world datasets are likely to be plagued by both missing data and unknown-but-complex topological diversity. Inspecting the spatial arrangement of a few diagnostic Clusterflock runs might yield some insight into the number of dominant clusters to expect, which would then inform algorithms that require estimates.

### In action: a recombination event in Staphylococcus aureus

In order to test our ability to detect and separate distinct populations of incongruent OGFs in biological systems, we used a well-known example of large-scale genomic recombination between two *S. aureus* clonal complexes (CC) [22]. The genomes of ST239 *S. aureus* appeared to have formed from a recombination event in which 20 % of a CC8 genome was replaced with the homologous portion of the genome from a CC30 strain. We chose 11 *S. aureus* strains (GCA_000146385.1, GCA_000012045.1, GCA_000011505.1, GCA_000011265.1, GCA_000013425.1, GCA_000204665.1, GCA_000159535.2, GCA_000027045.1, GCA_000017085.1, GCA_000236925.1, and SA21300), including examples from CC30, CC8 and ST239, and generated groups of orthologous genes across all their proteomes using orthologID [34]. The resulting sequence data matrix contained 2550 OGFs totaling 758,270 amino acid characters. Missing data was tolerated, but representation from at least four taxa for each gene was required.

Figure 2 shows three frames from the beginning of a 1000-frame simulation and three snapshots from even intervals thereafter. We have color-coded OGFs here for clarity: green maps to the hybridized portion of the genome, and red maps to the remainder. A complete video of this simulation can be found at https://youtu.be/v_4bDprmkpU.

**Fig. 6:**
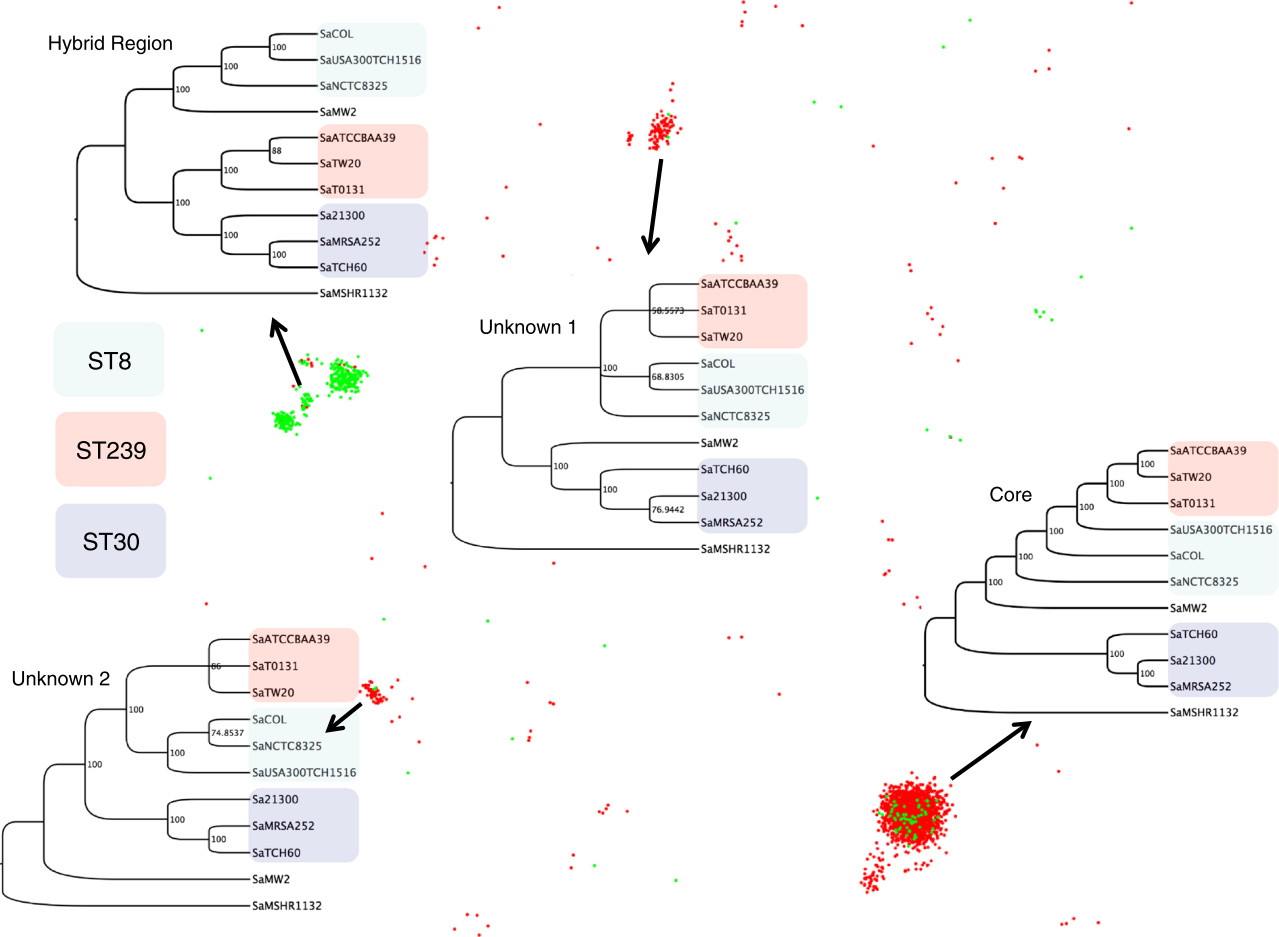
The final frame. Four parsimony trees corresponding to the four dominant flocks are shown superimposed on the final frame of the example simulation. Taxa are colored according to their phylogenetic group (MLST classification). The unknown phylogenies highlight genes that are members of two novel evolutionary histories

The sorting of OGFs by phylogeny was evident as early as the 10^th^ frame, and proceeded further as these initial seeds encountered one another in the virtual space. But hundreds to thousands of frames were required to amass flocks containing all congruent OGFs. The number of frames required was dependent on the general phylogenetic cohesion of the OGFs as well as their random starting positions and velocities in the virtual space. By the end of the simulation, the OGFs had self-organized in the leaderless manner characteristic of swarm behavior. Without having to estimate the number of expected evolutionary trajectories, we found that there were two dominant flocks: one corresponding to the recombined region, and the other to the rest of the genome. We observed two unexpected, smaller flocks (Fig. 6), the provenance of which did not trace to any known evolutionary event or functional class.

Because of the stochasticity inherent in this type of behavioral method, there was no guarantee that flocks of the recombined region or the rest of the genome would be complete: either or both might have entered the final frame in pieces. In other words, we need more statistical heft than one simulation can provide. To test the reproducibility of the flocks and identify robust flock membership, we repeated the simulation in parallel, randomly varying the initial position and velocity of each OGF. For the purposes of this example, we deployed 100 replicates.

**Fig. 7:**
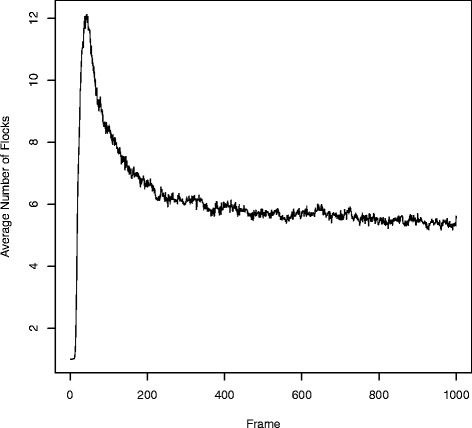
Auto-detected flocks per frame. Here, we show the average number of flocks detected at any given point along a 1000-frame simulation for the *S. aureus* simulation. The OPTICS spatial clustering algorithm was used to auto-detect flocks in the 100 replicate frames at each point along the simulation

Visual inspection of 100 final frames, or the thousands that may be desirable for other clustering problems, is prohibitive. We automated the analysis of each final frame using the ELKI [35] implementation of the OPTICS algorithm. Note that in contrast to our simulations, where we knew the number of clusters and could therefore leverage k-means clustering, we assume here that this number is unknown and, in the case of very complex datasets, unknowable. Figure 7 shows the average number of auto-detected flocks across all 100 simulations as a function of their frames. After an initial spike, or seeding stage, micro-clusters combined with neighboring micro-clusters that shared a congruent phylogenetic history. A near-exponential decay in the number of auto-detected flocks was followed by a steady state. In the case of *S. aureus*, by the 200^th^ frame, Clusterflock had isolated most of the evolutionary paths.

**Fig. 8:**
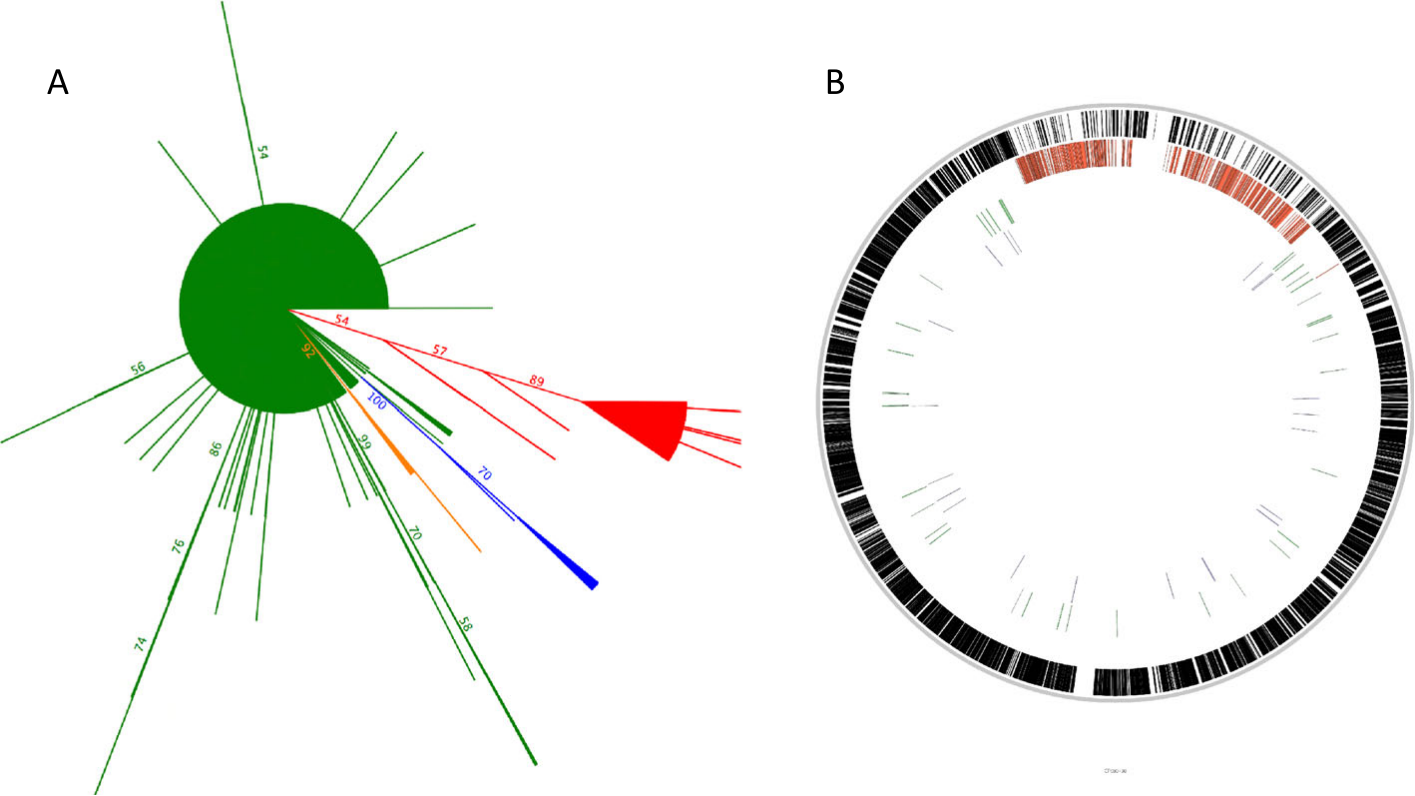
Consensus tree of *Staphylococcus aureus* flocks mapped to the USA300TCH1516 genome. **a** The neighbor joining the bootstrap consensus tree for 100 simulations is shown. The majority of the genome occupies the largest branch consisting of a virtual polytomy (*red*). Four other branches of note are highlighted, the largest of which describes the flock consisting of genes from the recombined region (*green*). **b** We constructed HMMs from the orthologous groups (genes) in each of the four dominant flocks and queried them against a USA300TCH1516 reference. Their genomic locations are shown in the four tracks displayed here. The outermost track is composed of genes from the largest flock. The second track localizes genes from the second largest flock to the known recombined region

We systematized flocking information by assigning a flock label to each OGF for each replicate. For example, replicate 1 might have resulted in five flocks; each of its 2550 gene families was therefore assigned to A, B, C, D, or E. Similar assignments were made across all replicates, and the labels were treated as character information in a matrix. The Flock Matrix therefore had as many rows as there were OGFs and as many columns as there were replicates. Organized in this way, we were able to derive our final sets of congruent OGFs using tree reconstruction, as shown in Fig. 8a. This topology highlighted groups of OGFs that regularly flocked together across all replicates. We used neighbor joining and assessed node robustness by bootstrapping 100 times. Because we had no external truth against which to measure success, we propose that the application of non-parametric bootstrapping to the Flock Matrix can serve as the basis for assessing validity [36–39]. We observed high levels of support for the flock composed of recombined genes and flocks that represented the two novel phylogenetic histories highlighted in Fig. 6.

**Fig. 9:**
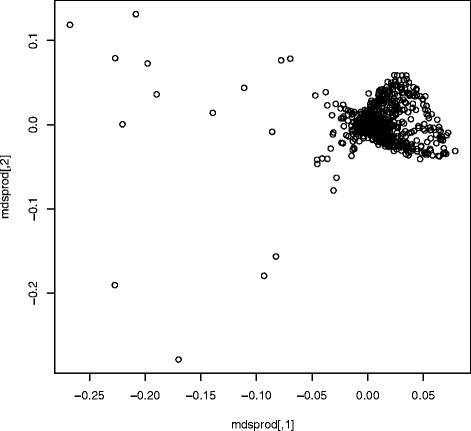
Multidimensional scaling of the *Staphylococcus aureus* dataset. We show a failed attempt to cluster the *Staphylococcus aureus* dataset with MDS. Most loci gathered in a single area, and we can see no separation between the recombined region and the rest of the genome

The main premise of this study was that we can use Clusterflock to detect the genes involved in the ST239 recombination event a priori by merely using pairwise interactions guided by incongruence metrics as OGFs encounter one another in a virtual space. There was no expectation that we would find only two unique flocks, an assumption required by other clustering methods keyed on partitional seeds. Indeed, two additional flocks emerged, with OGFs that tightly shared a unique historical signal distinct from our two main evolutionary classes. When we clustered these data with MDS (Fig. 9) there was no discernable pattern, a predicament most likely due to some combination of missing data and rate heterogeneity.

If, as we suspect, the conflicting histories of our two main flocks originated from the recombination event, we should observe the gene families’ sort based on the known boundaries of the structural change. By creating profile HMMs [40, 41] of each of our 2550 OGFs and mapping them to the *S. aureus* USA300/TCH1516 genome using hmmsearch in the HMMER package, we showed that the flock of OGFs corresponding to the recombined region (Fig. 6b), mapped there almost exclusively. The flock corresponding to the rest of the genome was enriched in genes outside the recombined region, but this enrichment was imperfect. Many genes from within the recombined region contaminated the flock representing the rest of the genome. The difference in length between these genes with respect to genes in the largest flock was zero, indicating that they were 100 % congruent with the non-recombined phylogeny. These select regions of the recombination event could have reverted through a series of subsequent recombination events, or may reflect the fact that the original recombination event did not replace one continuous section of the chromosome. A discontinuous pattern of recombination is known to occur in other bacteria [42, 43]. Other possibilities include convergent changes or high levels of conservation prior to the recombination event.

## Conclusions

Clusterflock is an enhanced version of Reynolds’ original flocking algorithm customized to function as a clustering technology. We have shown here that it is well-suited to isolating congruent gene families into discrete flocks, even if they have significant levels of missing data or rate heterogeneity. It can be used to identify a phylogenetic core of genes that share a vertical evolutionary signal while highlighting those that conflict in subtle ways. However the technique is general, and not restricted to evolutionary analysis [25]. Any distance metric scaled between 0 and 1 can be used to cluster any set of entities. In an era when supervised machine learning often captures the headlines in news on bioinformatics, Clusterflock is in the tradition of data mining: a bio-inspired clustering algorithm used to discover categories of entities without any training, any sense of the number of categories to expect, and any bias in how distant two entities must be to be considered distinct.

### Availability and requirements

• Project Name: Clusterflock

• Project Page: https://github.com/narechan/clusterflock

• Docker Hub: https://hub.docker.com/r/narechan/clusterflock-0.1/

• Operating System: Linux

• Programming Language: PERL

• Other Requirements: See manual in the distribution

• License: GPLv3
